# Lung ultrasound in young children with neurological impairment: A proposed integrative clinical tool for deaeration-detection related to feeding

**DOI:** 10.3389/fped.2022.932409

**Published:** 2022-07-27

**Authors:** Simona Fiori, Elena Moretti, Carolina Amador, Alice Martinelli, Rosa Teresa Scaramuzzo, Tiziana Controzzi, Roberta Battini, Luca Filippi, Andrea Guzzetta, Luna Gargani

**Affiliations:** ^1^Department of Clinical and Experimental Medicine, University of Pisa, Pisa, Italy; ^2^IRCCS Stella Maris Foundation, Pisa, Italy; ^3^Department of Neonatal Intensive Care Unit, Santa Chiara Hospital, Pisa, Italy; ^4^Institute of Clinical Physiology National Research Council of Italy (CNR), Pisa, Italy; ^5^Department of Surgical, Medical and Molecular Pathology and Critical Care Medicine, University of Pisa, Pisa, Italy

**Keywords:** cerebral palsy, lung ultrasound (LUS), neurological disorders, dysphagia, aspiration

## Abstract

Infants and children with neurological impairment, such as cerebral palsy (CP), often experience abnormal ingestion functions, including oropharyngeal dysphagia and gastroesophageal reflux disease, which led to aspiration-related respiratory complications, morbidity, hospitalization, or death. There is a lack of evidence-based, repeatable, infant-friendly instrumental procedures to assess aspiration-risk in infants with CP or other neurological disorders, with also a lack of clinical assessment measures to support the use of more invasive diagnostic techniques. To this purpose, in the current study we explore the feasibility of lung ultrasound (LUS), to assess lung deaeration possibly related to aspiration during meal, in a cohort of 35 subjects affected by CP or other encephalopathies, and 10 controls in the same age-range. We coupled LUS procedure with meal caregiver administration for each child. Our results support the feasibility of this innovative approach in the clinical setting. Exploratory findings revealed a number of lung abnormalities likely related to abnormal ingestion function in subjects. Subgroup analyses revealed possible differences in LUS abnormalities between CP and other encephalopathies, possibly related to different mechanism of disease or dysfunction. Also, some evidences arose about the possible relationship between such LUS abnormalities and feeding and swallowing abilities in CP or other encephalopathies. LUS showed preliminarily feasibility and effectiveness in detecting meal-related LUS abnormalities in a dynamic manner in the clinical setting. This approach demonstrated usefulness as a potential tool for improving assessment and management in complex care of infants and young children with severe neurological disorders.

## Introduction

Infants and children with neurological impairment commonly experience respiratory diseases, which represent a leading cause of morbidity and mortality that has barely altered in the last decades (1). Vulnerability to respiratory diseases is highly related to abnormal ingestion functions (WHO^1^), which is common in neurological impairment (1–3). Indeed, feeding and swallowing rely on the anatomical complexity at the pharynx crossroad between airways and digestive tract, whose coordinate neurophysiology is fundamental for functional deglutition (4). Among children with neurological impairment, cerebral palsy (CP) is the most common neurological disability (5), which manifests with a wide range in the severity of motor disorder and comorbid conditions, including swallowing disorders, communication, cognition, perception, seizures, and behavior (6).

Respiratory diseases and, in particular, pneumonia or recurrent respiratory illnesses are consistently major causes of morbidity, hospitalization or death in CP, with dysphagia, gastroesophageal reflux, and drooling being the principal manageable risk factors for silent or overt aspiration (1, 2, 7). There has been a recent effort to reach a consensus for the prevention and management of lung acute or chronic diseases in young people with CP, with identification of risk being based on history and multidisciplinary assessment of physical examination, eating and drinking abilities, and instrumental assessment (1), as dysphagia is considered a major contributor to respiratory complications. Several studies showed varying prevalence of dysphagia in CP, varying from 40 up to 90% independently from motor or oral impairment classification (8–10), thus giving a dimension of the potential respiratory risk in such a population. Moreover, younger ages, gross motor function classification system (GMFCS) IV or V, weekly respiratory symptoms and asthma were associated with repeated antibiotics use and respiratory hospitalization in CP, confirming that individuals with oromotor dysfunction are more likely to have serious respiratory illness (2, 11), which is mostly related to aspiration. Aspiration of liquids, solids, saliva, or refluxate due to abnormal swallowing determines inflammation, or infection for bacteria in the aspirate, and are thought to be the main contributors to the abovementioned respiratory complications.

Aspiration in infants and children with dysphagia is a common condition also in encephalopathies of other origins that CP, which result in severe neurological impairment, with varying prevalence up to 80% (12–14). Moreover, dysphagia in children with other neurological conditions other than CP is poorly documented, with no shared transversal pathways to improve the clinical approach of their complex care.

Tracheal aspiration has been documented by Videofluoroscopic Swallowing Study (VFSS) in about 30% of children with CP, with 20% being silent (8). Despite VFSS being considered the reference standard for aspiration detection, it has to be noticed that videofluoroscopy reflects only a snapshot of swallowing, with limited estimation of the impact of fatigue, pacing, positioning during the full meal (15). Also, rheological properties of the barium-impregnated thickened and unthicken liquids may not reflect the real characteristics of the children usual meal, thus making the clinical interpretation of the radiological results, and consequent clinical recommendations, sometimes uncertain (13, 16). This is also in support of the postulation that VFSS may not detect all children who aspirate daily (17). The repetition of an unsatisfactory VFSS assessment is however unlikely in the clinical practice, due to its radiological and technical issues that makes VFSS poorly repeatable in the same child. Fiber-optic endoscopic examination of swallowing (FEES) is another option for exploring deglutition and aspiration, however, as VFSS, it focuses on a single study and, mostly due to low comfortability, it is also poorly reproducible in the same patient over a limited time and for monitoring intervention effectiveness, especially in infants and children. Finally, there is a lack of consistent and reliable standards for instrumental assessment of aspiration risk in children with dysphagia (13).

Approaches to diagnosis and intervention in general in infants with neurological disabilities has moved toward very early stages of development for targeting plasticity with interventions. Despite some aspects of clinical picture of infants with neurological impairment, in particular in CP, can be indeed early assessed and prognosticated, similarly to GMFCS, in a longitudinal study Benfer et al. describe the progression of oropharyngeal dysphagia in a cohort of pre-school infants with CP by using valid and reliable clinical tools. Interestingly they noticed a reduction of prevalence of dysphagia symptoms between 18 to 24 and 30 to 36 months of age, which was attributed to a developmental limitation of ingestion functions at early ages (18). These reports suggest an evolutionary aspect of dysphagia in CP infants that should bring to implementation of infant friendly and biological risk-free, thus repeatable, diagnostic tools to support early assessment and monitoring, by reducing the risk of inappropriate use of invasive instrumental tools with no risk underestimation.

Lung ultrasound (LUS) is a non-invasive, radiation-free tool for the diagnosis of acute and chronic pulmonary conditions in both infants and adults, with high sensitivity and specificity (19–21). Due to its sonographic nature, it is infant-friendly, especially at younger ages when biological tissues are more susceptible to radiation exposure (22). Air/fluid ratio is responsible for LUS findings that reflect lung aeration. Several semi-quantitative LUS scores have been validated against oxygenation levels (23), computed tomography findings (24) or mild to moderate inflammation (25), demonstrating validity of this approach for the study of both infection and inflammation processes.

The present cross-sectional study aimed at studying the feasibility of assessing meal-related LUS abnormalities in a cohort of infants aged 6 months–4 years with neurological disorders and in a sample of age-matched controls. In addition, we aimed at: (i) comparing the load of identified LUS abnormalities between infants with neurological disorders and controls; (ii) evaluating the relationship between the clinical feeding and swallowing assessment and LUS meal-related abnormalities in neurologically diseased infants; and finally (iii) assessing the impact of the nature of the neurological disorder on the clinical feeding and swallowing and LUS assessment by comparing infants with CP and infants with other disorders than CP within the whole cohort.

## Materials and methods

### Subjects

For this perspective study, infants with neurological disorders were recruited at Stella Maris Scientific Institute, Neurology Section, between January 2020 and June 2021.

Inclusion criteria for this study were: (1) infants within an age range 6 months–4 years; (2) risk of- or a diagnosis of- CP or other encephalopathies with abnormal muscle tone; and (3) risk of abnormal ingestion function, including dysphagia or gastroesophageal reflux disease (GERD). As exclusion criteria we considered having (1) primary lung disorders, related or not to the neurological condition; and (2) disorders of consciousness, related or not to the primary neurological disorder. Age-matched typically developing children were recruited as controls. The institutional review board approved the study (Pediatric Ethics Committee of the Tuscany Region, study opinion registration number: 107/2019) and informed parental consent was obtained for all participants.

### Lung ultrasound

Lung ultrasound exam were performed with a portable machine with a linear probe (9–12 MHz) with specific setting for infants (Lumify, Philips Medical Systems, Andover, MA, United States). Acquired LUS images and movies were saved and available for off-line for analyses. A previously described scanning scheme for the systematical assessment of lung parenchyma in children was applied (26). In detail, the thorax was divided into three major scanning areas (anterior, lateral, and posterior) divided by the parasternal, anterior axillary, and posterior axillary anatomical lines on both the right end the left hemithorax. Previous literature on LUS supports the usefulness of a quantitative LUS approach (27), with different scoring systems showing different accuracy across several pulmonary conditions. As this is the first study applying LUS to this specific condition, a conservative approach to LUS findings reporting and scoring was applied. In particular, a deaeration score was used to classify LUS findings, by considering *a priori* the following signs in each scanning area: the number of B-lines; presence of small peripheral sub-pleural consolidations; presence of large consolidations. In detail, B-lines are defined as discrete laser-like vertical hyperechoic reverberation artifacts that arise from the pleural line and extend to the bottom of the screen without fading, moving synchronously with lung sliding. We defined consolidations in presence of a subpleural echo-poor region or one with tissue-like echotexture, with 10 mm for the longitudinal diameter as a cut-off between small and large (28). The raw number of B-lines yielded the B-lines score, while small consolidations received a score of 5 and large consolidations received a score of 8 and converged in the Consolidation score. The global LUS score for each assessment resulted by the sum of the B-line and Consolidation scores. When consolidations were detected, a further LUS evaluation was repeated after 3 hours to check for consolidation involution.

To the purposes of this study, LUS was coupled to a feed trial as follows. Each infant performed a baseline LUS assessment before meal administration (pre-meal LUS assessment), with the relative abnormality score assigned off-line. The LUS assessment was repeated within 15 minutes after the end of the meal (post-meal LUS assessment), with score assignment off-line. In case of consolidation, the LUS assessment was repeated 3 hours after the post-meal assessment. In order to characterize the representative feeding and swallowing findings with respect of patients’ age/neurological condition, it has been asked the caregivers to provide a typical administered meal with usual consistencies. In order to systematically evaluate and quantify off-line the feeding and swallowing clinical competences of each infant, the meal administration was video-recorded.

### Clinical feeding and swallowing assessment

#### Development of the clinical feeding and swallowing assessment tool

As no published data collection or clinical measure existed to absolutely determine the risk of aspiration in this population, our choice of measures was based on research literature concerning feeding difficulties in CP and other clinical populations and the clinical expertise of the multi-disciplinary research team. Literature was searched for clinical measures for the assessment of feeding and swallowing abilities in infants and preschool children with neurological impairment (9, 29). A dedicated tool was developed to systematically assess the feeding and swallowing abilities in the clinical evaluation.

#### Pilot testing and discussion

Suitability of the developed tool was assessed through pilot testing in three case-series of video-recorded feeding observations for subjects with neurological impairment, including CP and other encephalopathies, among patients referred at Stella Maris Scientific Institute. After the case-series evaluation, the main contents and the possible scoring procedure for the feeding and swallowing tool were discussed among a multidisciplinary team including child neurologists, speech and language pathologists, pediatricians with an experience in the field of feeding and swallowing difficulties related to pediatric neurological impairment. Subsequently, the item description and scoring procedure were revised according to inputs from the pilot testing. A pilot interrater reliability of the proposed tool was performed in 15 children with neurological impairment by three raters with different expertise (experienced tertiary care SLP, experienced healthcare SLP, and post-graduate SLP) for the final paradigm of the proposed scale to be applied to the current group of subjects.

#### Description of the clinical assessment and relative scoring calculation

The clinical assessment tool for feeding and swallowing abilities includes three sections, corresponding to oral-motor competences: sucking, puree/semi-solid/solid feeding, and drinking. Each section includes a number of items that are assessed on the video-recording of the typical meal of the subject, administered in an ecological manner. Child participation, oral-motor competences and unsafe swallowing alerts are systematically assessed. For each single item is given a score of 1 if abnormal function is observed. Each item is summarized to result in a Section score. The sucking section includes seven items; the feeding section eight items and the drinking section includes five items. Only competences that are directly assessed, are included in the score. The section scores are summarized and converted in a percentage score, where a higher percentage correspond to a higher degree of impairment. The summary of the Section score is the total feeding and swallowing abilities score, which is calculated as the weighted percentage of the assessed competences. Inter rater reliability was tested in 35 subjects among three raters. Overall, interrater reliability coefficients of the three Sections and of the Total scores varied between 0.82 and 0.94 (very good to excellent) (unpublished data, in preparation) (30).

### Statistical analysis

All subjects completed the LUS monitored meal. For both subjects and controls, pre-meal and post-meal LUS abnormality scores and the difference between pre-meal and post-meal assessment (delta-LUS) were calculated for each LUS score (B-line, Consolidation, and global LUS) and included in the analyses. For the clinical evaluation, the total feeding and swallowing assessment score (FSs) was included in the analyses.

An independent sample Mann–Whitney U Test was performed to assess differences between pre-, post-meal and delta LUS abnormalities between subjects and controls. An independent-samples Kruskal–Wallis Test was used to determine differences among CP, other encephalopathies and control groups for LUS meal-related measures (pre-meal and post-meal), with *post hoc* pairwise comparisons were performed to correct for multiple comparisons (31).Non-parametric related-sample Wilcoxon signed rank test was used to determine differences between subjects and controls and between CP and other encephalopathies for pre-meal and post-meal LUS abnormalities. Non-parametric one-tail Spearman’s rho was applied to assess the relationship between the clinical score (FSs) and post-meal and delta LUS abnormality scores.

Statistical analyses were performed using SPSS version 26. Results were considered significant at *p* < 0.05.

## Results

**FIGURE 1:**
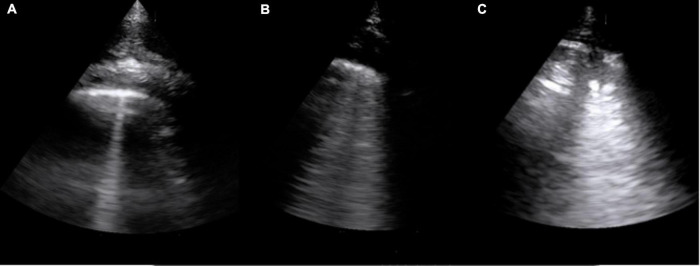
Example of most significant findings in a subject from the CP group. **(A)** B-line; **(B)** confluent B-lines; and **(C)** consolidation.

Thirty-five infants (mean age 20.2 months, range 6–51 months) with neurological disorders and ten normally developing controls (mean age 20.4 months, range 9—48 months) were recruited for this study. Based on neurological diagnosis children were classified in subgroups of CP (*n* = 21 subjects) or other encephalopathies (*n* = 14 subjects). Demographic, clinical, and LUS scores information for each group are provided in Tables 1, 2. Examples of LUS findings are provided in Figure 1.

**TABLE 1:** Demographics and clinical characteristics.

Demographics and clinical characteristics	
Age, median (IQR) (months)	20, 17 (6–51)
Sex (males/total)	21/35
Cerebral palsy, CP (*N*/total)	21/35
**GMFCS level**	
I (*N*/total)	1/21
II	0/21
III	3/21
IV	6/21
V	11/21
**Primary motor type**	
Spasticity (*N*/total)	7/21
Dyskinesia	4/21
Spastic-dyskinetic	7/21
Hypotonia	3/21
Other encephalopathies	14/35
Genetic disorder	5/14
Epileptic encephalopathy	3/14
Neurotransmitter disorder	2/14
Pelizaeus-merzbacher disease	1/14
Mitochondrial disease	2/14
Tay-Sachs disease	1/14
**Primary motor type**	
Spasticity (*N*/total)	2/14
Dystonic-dyskinetic	1/14
Spastic-dystonic	1/14
Hypotonia	10/14

IQR, Inter quartile rank; N, number; CP, cerebral palsy; GMFCS, Gross Motor Function Classification System.

**TABLE 2:** Lung ultrasound and clinical findings.

	Cerebral palsy	Neurological disorders	Controls
Pre-meal LUS (median, range)	4, 13	4, 19	0, 1
Post-meal LUS (median, range)	7, 22	4, 19	0, 1
Delta LUS (median, range)	−2, 18	−0.5, 10.5	0, 0
Feeding history (median, range)	57, 86	42, 58	na
Feeding and swallowing (median, range)	46, 100	50, 86	na
Composite (median, range)	53.5, 85.5	47.5, 72	na

LUS, lung ultrasound.

There were significant differences between subjects and controls among all meal LUS-related measures. In particular, subjects have higher pre- (*p* < 0.001), post-meal (*p* ≤ 0.001) and delta (*p* < 0.001) LUS scores, corresponding to more abnormalities, compared to controls. Significant differences in LUS abnormality scores were also detected among the three subgroups of CP, other encephalopathies and controls, with *p* = 0.026 for pre-meal LUS score and *p* = < 0.001 for post-meal LUS score. *Post hoc* analysis revealed that pre- and post-meal LUS score were significantly higher in CP (pB = 0.03 and pB = 0.004, respectively) and other encephalopathies (pB = 0.05 and pB < 0.001, respectively) compared to controls. At Wilcoxon paired test, a significant difference between pre- and post-meal LUS abnormalities was found in the subjects’ group (*p* = 0.002), whilst no difference emerged in controls. In subjects, B-line score and Consolidation score showed differences between pre- and post-meal when evaluated separately, with *p* = 0.012 and *p* ≤ 0.001 respectively. When subgroup analysis was performed, the difference between pre- and post-LUS abnormalities remained consistent only in the CP group compared to other encephalopathies, with *p* = 0.006 for global LUS score, *p* = 0.02 for B-line score and *p* = 0.007 for Consolidation score. A trend toward significancy resulted for differences between pre- and post-meal Consolidation score in the group of other encephalopathies (*p* = 0.83).

In infants with neurological disorders, the FSs showed a correlation with global post-meal LUS (*p* = 0.02, rho = 0.34) and delta LUS (*p* = 0.01, rho = −0.38) scores, with a weak-moderate strength of the association (32). The FSs moderately correlated with delta Consolidation score (*p* = 0.001, rho = −0.5 and *p* = 0.008, rho = −0.41, respectively), whilst no relationship emerged with B-line score. In the subgroup analyses, in the CP group the FSs moderately correlated with global post-meal LUS score (*p* = 0.026, rho = 0.43) and delta LUS score (*p* = 0.038, rho = −0.40). No relationship resulted in the group of other encephalopathies. The FSs correlated with the delta Consolidation score (*p* = 0.02, rho = −0.46) in CP subjects. In other encephalopathies subjects, the FSs strongly also correlated with the delta Consolidation score (*p* = 0.002, rho = −0.72 and *p* = 0.002, rho = −0.71, respectively).

## Discussion

The present study supports the feasibility of assessing meal-related LUS abnormalities in a cohort of infants aged 6 months–4 years with neurological disorders and in a sample of age-matched controls. To the best of our knowledge, this is the first study that include LUS monitoring to the feeding trial, in order to assess silent or overt aspiration related to meal in infants and young children with neurological impairment and healthy controls. Thanks to its high sensitivity and specificity compared to radiological chest imaging (19, 28, 33), LUS is a widely recognized technique for assessing lung aeration and has demonstrated its usefulness in point-of-care diagnostics both for adult and pediatric populations (25, 28, 34, 35). Also, LUS demonstrated a significant impact for therapeutic management (27, 36) and reduction of X-ray exposure for patients (37), which is even more relevant at early developmental ages (22).

Lung ultrasound demonstrated meal-related abnormalities in infants with neurological disorder, while no significant abnormality was detected in our sample of typically developing controls. In infants with neurological impairment LUS detected both pre- and post-meal abnormalities, with the latter to a larger extent. This is the first evidence of the potential sensitiveness of this technique to detect silent or overt aspiration related to swallowing disorders in infants and young children with neurological impairment. The exact clinical correlate of these lung findings may be only hypothesized. Several evidences support the contribution of feeding and swallowing difficulties to pulmonary acute and chronic disease in children with neurological impairment (3, 11, 15). Indeed, our results support a chronic (pre-meal) and contingent (post-meal) lung involvement in neurological disorders. Despite several factors might contribute to baseline, chronic, lung abnormalities (e.g., chest hypomobility), the post-meal increase in LUS findings support at least one, but detectable, contribution to meal-related aspiration. In order to verify the possible impact of mobility on pulmonary abnormality, at *post hoc* we explored the relationship between GMFCS (38) and LUS pre-, post-meal, or delta findings in the CP subgroup, but no relationship emerged, consistently with recent findings in infancy (39), conversely with respect to older ages (40).

As the use of X-ray is cautionary in the pediatric population, only limited evidence supports the exact rate of aspiration in children and its relationship with acute or chronic complications, such as inflammatory or infection-based pneumonia. VFSS is the radiological reference standard for aspiration/penetration assessment, however, beyond X-ray that limits its repeatability especially in the pediatric population, VFSS requires a certain degree of cooperation of the infant/child; there is also a lack of shared procedures, with possible limited evidences due to the rheological properties of the barium-impregnated thickened or unthicken liquids compared to the infant’s typical meal and the fact that an exam only reflects one or a few deglutition acts (13). For all these reasons, despite being conclusive for more severe cases, mostly when overt aspiration occurs, the VFSS assessment may not give an idea of the chronic, instead silent, aspiration risk of a child with neurological impairment (13, 17). Moreover, silent aspiration is thought to be the majority in infants and children with feeding and swallowing disorders (39–41). Our results support the introduction of LUS as a non-invasive, infant-friendly, handy, repeatable bed-side and “arm-side” tool to assess aspiration risk in pediatric dysphagia. The way LUS is introduced in this study is in line with its conception of being an extension of a clinical, feeding and swallowing, examination (34, 42). Indeed, we propose LUS information as a possible add-on value to the clinical feeding evaluation, not as an alternative to more complex radiological approach that are expected to answer well-defined and argued clinical questions. The on-line LUS meal-related assessment has the advantage of providing dynamic information about possible risk and safety of feeding and swallowing in a completely ecological manner in the clinical setting. The introduction and evaluation of this proposed approach is needed as there is a lack of evidences about the accuracy of the clinical feeding evaluation in detecting oropharyngeal aspiration in children (13) and because of the abovementioned limitations of the radiological approaches. The potential repeatability of this LUS tool, and the integration of information from other studies when needed, including VFSS or FEES, may also help to differentiate the risk related to primary (drink/food, that can be assessed separately) or secondary (gastroesophageal reflux) aspiration. Moreover, LUS has the potential of improving feeding and swallowing difficulties management at early critical ages of development in particular for infants with CP, when functional trajectories that includes development of ingestion function is still in evolution (18). Further studies are mandatory to assess the clinical impact of LUS findings on respiratory status and quality of life of infants with feeding and swallowing disorders risk due to neurological impairment since very early stages of their development. Despite LUS being a technique that has been proven to be easy to learn also by ultrasound non-expert care providers (43–45), it will be fundamental to further test this feasible protocol to understand reliable cut-off values for abnormalities and identification of risk categories among patients with neurological disorders at different ages and according to their clinical characteristics.

Most literature on dysphagia in neurologically impaired children do not consider systematically the nature of the neurological disorder as a primary condition for estimating the risk of aspiration (3, 17, 46). In our sample we included infants and children with CP and other encephalopathies. Indeed, our result support possible differences in the *a priori* risk of aspiration due to the nature of the neurological disease. Subgroup analyses were consistent with whole group analyses, as each CP and other encephalopathies group showed significantly higher pre- and post-meal LUS abnormalities compared to controls. A significant difference between pre- and post-LUS abnormalities remained consistent only in CP, thus supporting the fact that infants with CP show more consistently a significant increase in LUS abnormalities after meal administration. However, despite a number of pre-meal abnormalities was present, no significant increase after meal emerged in subjects with other encephalopathies, with the exception of a trend for findings of lung consolidations. CP is a complex disorder mostly of movement and posture, which determines altered development of physiological processes due to congenital brain injury. So, it is not surprising that the pathophysiology of the disease may influence the risk of respiratory complications compared to other encephalopathies. This supports the need for personalized approaches to assessment and care, and the need for valuable and objective diagnostic tools, such as LUS might be whether these preliminary encouraging findings should be confirmed in larger cohorts and longitudinal studies.

As the last aim of the current study, some analyses were conducted to evaluate the impact of systematically assess aspects of the feeding and swallowing history and of the clinical assessment procedure on post-meal LUS abnormalities. Despite clinical feeding observation has been reported in general as having lower accuracy in detecting aspiration (13), we explored the possible relationship between some aspects of the clinical feeding and swallowing assessment of these subjects and post-meal and delta LUS findings. Indeed, our results support the hypothesis that more severe meal-related LUS abnormalities correlated with worse clinically directly observed feeding and swallowing function. In particular, our findings reveal more severe impairment of feeding and swallowing abilities observed directly being correlated to global both post-meal and delta LUS abnormalities, with a weak-moderate strength of the association (47). When exploring the independent contribution of different LUS findings to clinical assessment, consolidations demonstrated a moderate correlation with clinical measures, whilst no correlation emerged for B-lines assessment in the whole group of subjects with neurological impairment. When the relationship between LUS and feeding and swallowing clinical measures was explored in the subgroups of subjects with CP and other encephalopathies, correlation remained consistent in the CP group only between direct feeding and swallowing assessment and global LUS abnormalities. Interestingly, while no relationship emerged among B-line assessment and any of the clinical measures, consolidation assessment showed a moderate correlation with direct clinical assessment in CP subjects. Furthermore, in subjects with other encephalopathies than CP, the direct clinical assessment showed strong correlations with LUS findings. Remarkably, when subgroup analyses for CP and other encephalopathies are performed by parallel analyses of relationships between clinical assessment and LUS, while the correlations described for the whole group remained consistent in the CP group, strong relationship emerged in the group with other encephalopathies between consolidation assessment and direct clinical observation. Despite other than CP, the group of other encephalopathies is however heterogenous in its pathogenesis, so no inferences can be made on mechanisms of ingestion function difficulties in this group. Taken together, these findings support the need of better defining the LUS meal-related profile depending on neurological disorders. Despite assessing B-lines and consolidation shows some advantages in the CP group, most consistent results seem related to consolidation assessment.

This study provides preliminary evidences to encourage the use of LUS as an integrative tool to assess feeding-related lung abnormalities in the complex care of infants and toddlers with neurological disorders. Further studies are mandatory to verify the utility of this proposed feasible tool at medium/long-term and its value as an add-on resource to current clinical and instrumental protocols. Our study has some limitations. Despite a certain amount of lung abnormalities is detected by our approach, we cannot exclude the coexistence of a number of aspiration events that do not determine detectable consequences on lungs based on LUS. Also, no patient with primary lung disorders was included in this preliminary study; inclusive cohorts are mandatory to guarantee the generalizability of the proposed LUS feeding-related approach to subjects with primary pulmonary diseases. Further, only children older than 6 months have been included in the current study; as maturational factors may impact feeding and lung at younger ages (48, 49), specific cohorts are required to extend the applicability of this LUS approach. Finally, we propose here the potential advantage of detect aspiration during the typical infant/toddler meal, to support the evaluation of effective infant daily risk; however, as a potential implementation of this proposed approach, defined types and consistencies of administration may support the standardization of the procedure in future studies.

## Data availability statement

The raw data supporting the conclusions of this article will be made available by the authors, without undue reservation.

## Ethics statement

The studies involving human participants were reviewed and approved by the Pediatric Ethics Committee of the Tuscany Region. Written informed consent to participate in this study was provided by the participants or their legal guardian/next of kin.

## Author contributions

SF, EM, RS, TC, and LG contributed to the study conception, design, data acquisition, and manuscript drafting. CA and AM contributed to the study design and data acquisition. LF, RB, and AG substantively revised the study design and the drafted manuscript. All authors approved the submitted version of the manuscript.

## Footnotes

1
http://apps.who.int/classifications/icfbrowser/
